# Novel Essential Oils Blend as a Repellent and Toxic Agent against Disease-Transmitting Mosquitoes

**DOI:** 10.3390/toxics11060517

**Published:** 2023-06-08

**Authors:** Chinnaperumal Kamaraj, Rajappan Chandra Satish Kumar, Khalid A. Al-Ghanim, Marcello Nicoletti, V. Sathiyamoorthy, Sabarathinam Sarvesh, Chinnasamy Ragavendran, Marimuthu Govindarajan

**Affiliations:** 1 Interdisciplinary Institute of Indian System of Medicine (IIISM), Directorate of Research, SRM Institute Science and Technology, Kattankulathur 603 203, Tamil Nadu, India; dean.iiism@srmist.edu.in (R.C.S.K.); sarveshs1@srmist.edu.in (S.S.); 2 Department of Zoology, College of Science, King Saud University, Riyadh 11451, Saudi Arabia; kghanim@ksu.edu.sa; 3 Department of Environmental Biology, Foundation in Unam Sapientiam, Sapienza University of Rome, 00185 Rome, Italy; marcello.nicoletti@uniroma1.it; 4 Ayurvedic Manufacturing, Kancheepuram 631 501, Tamil Nadu, India; 5 Department of Conservative Dentistry and Endodontics, Saveetha Dental College and Hospitals, Saveetha Institute of Medical and Technical Sciences (SIMATS), Chennai 600 077, Tamil Nadu, India; ragavan889@gmail.com; 6 Unit of Vector Control, Phytochemistry and Nanotechnology, Department of Zoology, Annamalai University, Annamalainagar 608 002, Tamil Nadu, India; 7 Unit of Natural Products and Nanotechnology, Department of Zoology, Government College for Women (Autonomous), Kumbakonam 612 001, Tamil Nadu, India

**Keywords:** essential oil, mosquitoes, larvicide, repellent, molecular docking

## Abstract

Bio-insecticidal research has focused on long-term vector control using essential oils (EOs). This study examined the larvicidal, oviposition-deterrent, and repellent properties of five medicinal herb-based EO formulations (EOFs) on mosquitoes that are vectors of dengue, filariasis, and malaria. EOFs were significantly more toxic to the larvae and pupae of *Culex quinquefasciatus*, *Anopheles stephensi*, and *Aedes aegypti* with LC_50_ = 9.23, 12.85, and 14.46 ppm, as well with 10.22, 11.39, and 12.81 ppm, with oviposition active indexes of −0.84, −0.95, and −0.92, respectively. Oviposition-deterrent repellence was found in 91.39%, 94.83%, and 96.09%. EOs and N, N-Diethyl-3-methylbenzamide (DEET) were prepared at various concentrations for time duration repellent bioassays (6.25–100 ppm). *Ae. aegypti*, *An. stephensi*, and *Cx. quinquefasciatus* were monitored for 300, 270, and 180 min, respectively. At 100 ppm, EOs and DEET had comparable repellence in terms of test durations. EOF’s primary components d-limonene (12.9%), 2,6-octadienal, 3,7-dimethyl, (Z) (12.2%), acetic acid, phenylmethyl ester (19.6%), verbenol (7.6%), and benzyl benzoate (17.4%) may be combined to make a mosquito larvicidal and repellant equivalent to synthetic repellent lotions. In the molecular dynamics simulations, limonene (−6.1 kcal/mol) and benzyl benzoate (−7.5 kcal/mol) had a positive chemical association with DEET (−6.3 kcal/mol) and interacted with the OBP binding pocket with high affinity and stability. This research will help local herbal product manufacturers and the cosmetics industry in developing 100% herbal insect repellent products to combat mosquito-borne diseases, including dengue, malaria, and filariasis.

## 1. Introduction

Mosquito vectors (Arthropoda) remain one of the major threats to community safety worldwide. They act as vectors of multiple pathogenic viruses, protozoa, and nematodes, responsible for numerous dangerous diseases [1,2,3]. Mosquito-borne illnesses cause around 2 million fatalities annually, which are widespread in over 100 countries and threaten up to 2.1 billion people globally. In India, which has a population of 1.4 billion, at least 1 million children each year die from diseases spread by mosquitoes [4,5]. The emergence of mosquitoes in numerous Indian towns results from rapid urbanization and industry without adequate drainage infrastructure [6].

The transmission of diseases is caused by infected female mosquitoes, including dengue fever, malaria, filariasis, Japanese encephalitis, West Nile fever, and Zika. Dengue, chikungunya, zika, and yellow fever are all spread by *Aedes aegypti* mosquitoes [7], Wuchereria is spread primarily by *Culex quinquefasciatus* mosquitoes [8], and malaria is spread mainly through *Anopheles stephensi* mosquitoes [2]. The only known way to prevent or control these deadly illnesses is via efficient vector management [9,10,11]. This is due to the lack of a clinically relevant therapy or vaccination. Synthetic insecticides have been used to successfully interrupt the insect-borne illness transmission cycle. However, massive and repeated use of these chemicals reduces their efficacy and leads to environmental problems, such as mosquito resistance, ecological disruption, and negative effects on mammals [12,13,14]. The search for innovative insecticidal chemicals has been sparked by the negative consequences of synthetic insecticides and mosquito tolerance [15,16].

When applied to human skin, insect repellent lotions (also known as “chemically designed mosquito repellents”) are combinations of synthetic or natural substances that make mosquitoes avoid the treated individual [17]. Synthetic DEET, an extremely active chemical against blood-sucking mosquitos and other insects, is a common ingredient in insect repellent sprays and lotions [18]. In addition to the increasing cost, its ongoing usage can cause hazardous reactions in people, particularly youngsters and aged persons [19]. Alternatively, essential oils (EOs) are plant-based natural mosquito repellents that could replace chemical repellents to prevent any potential side effects [19].

Compared to synthetic repellents, they are regarded as harmless and environmentally friendly [17]. We employ the cutting-edge “Herbal Mosquito Repellent Oil” (HMRO), with insecticidal characteristics derived from herbal EOs, to test its control effect on different mosquito genera. HMRO is a herbal preparation made from the combination of five medicinal plants, namely *Eucalyptus globulus* (Leaves), *Cymbopogon nardus* (Leaves), *Citrus limon* (Fruit peel), *Trachyspermum ammi* (Seeds), and *Mentha arvensis* (Areal parts). The current research examines the larvicidal, pupicidal, ovicidal, oviposition-deterrent, and repellent properties of EOs from native plants growing in the Western Ghats of South India. Plant-based extracts and EOs have been shown to be highly relevant in previous studies. Five different plant species’ aerial parts, seeds, and peels were utilized to obtain their EOs via steam distillation [19,20]. Mosquito toxicity bioassays on in vitro cultured eggs revealed that herbal EO formulations impacted the larvae, pupae, and adults of the vectors transmitting dengue, malaria, and filariasis diseases, notably *Ae. aegypti* (AA), *An. stephensi* (AS), and *Cx. quinquefasciatus* (CQ). The bioactive components of EOs were analyzed using gas chromatography–mass spectrometry (GC-MS). Additionally, seven main constituents from herbal essential oil formulations were used as a starting point for combining ligand- and structure-based molecular docking techniques against mosquito odorant-binding proteins (OBPs4). We performed structural and pharmacophores-related findings to identify possible natural products from the EOF, using as a reference the crystallographic structure of DEET interacting with the OBP4 homodimer of *Anopheles gambiae* (Agam-OBP4) PDB: 3Q8I. Our findings may help in the development of new repellents with increased affinity and discernment to the protein binding compact. They may also provide insight into the process by which these substances work to prevent insect olfactory identification. From our perspective, this is the first study to use in silico techniques on a formulation of herbal EOs and to evaluate the potential of five different plant chemicals as mosquito OBP4 inhibitors. EOFs could be used to commercialize a 100% herbal “Mosquito Repellent Oil” to prevent mosquito-borne illnesses.

## 2. Materials and Methods

### 2.1. Collection and Identification of Plant Material

Fresh leaves from *Cymbopogon nardus* (L.) Rendle (Poaceae), *Eucalyptus globulus* Labill, 1800 (Myrtaceae), *Mentha arvensis* L. (Lamiaceae), fruit peel from *Citrus limon* L. (Rutaceae), and dried seeds from *Trachyspermum ammi* L. (Apiaceae) were acquired between July and September-2022 from the Jawadhu Hills in the Tiruvannamalai District of Tamilnadu, India. Dr. C. Hema, Head of the Department of Botany, validated the taxonomic identity of these plants. The freshly collected plant material was frozen at −20 °C before being used for the extraction of herbal EOs.

### 2.2. Extraction of Essential Oils (EOs)

**Figure 1:**
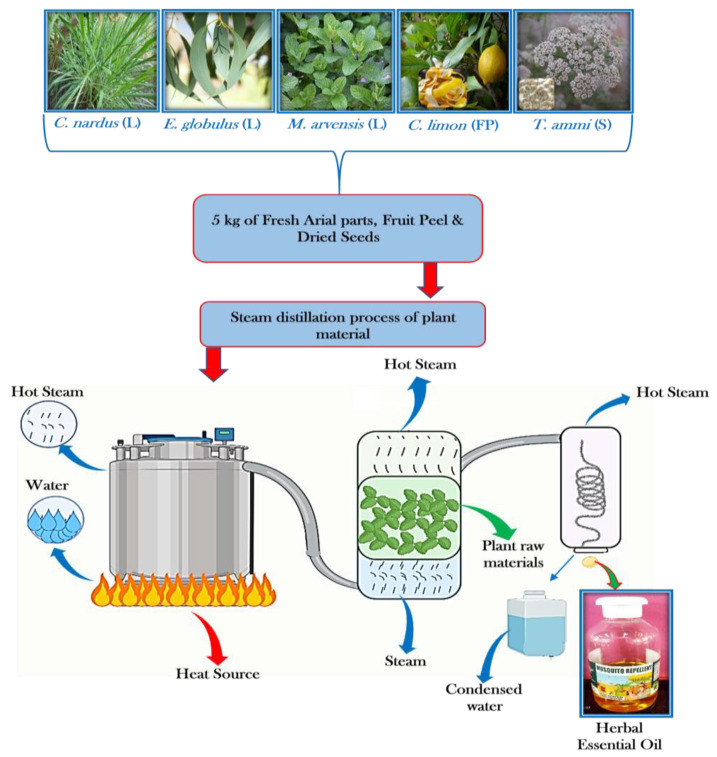
The process for extracting essential oils is depicted in a flow chart.

EOs were attained from the leaves, fruit peels, and dried seeds of the collected selected plants using the steam distillation method [20,21]. Small leaves and fruit peels were chopped with a sharp knife, and crushed seeds were used. Utilizing a stainless steel distillator, the weighted plant material was put through steam distillation (5 kg). The bottom of the stainless-steel tank was filled with 5 L of deionized water to keep it from contacting the plant parts, which were placed inside a sieved partition, and the vessel was heated on an electric hotplate. A water condenser reduces the heated vessel’s steam. The EOs were removed from the upper coating while the residue was separated via decantation. Utilizing (100 mL) HPLC grade *n*-hexane solvent, liquid–liquid extraction (LLE) was carried out on the residual distillate. The acquired solvent coatings were collected, dried with anhydrous MgSO_4_, and filtered, and a rotary evaporator removed the solvent at 25 °C and lower pressure. LLE was collected, then the essential oil was combined and weighed. Figure 1 depicts the flowchart of the extraction process. The obtained EOs were kept in a glass container and kept at a temperature of −20 °C until use.

### 2.3. Culturing of Mosquito Species

Early third instar larvae of AA, AS, and CQ were provided by Dr. J.A. Justin, State Entomology Consultant. The larvae were then maintained at room temperature under a 14:10 (L:D) photoperiod maintained in plastic vessels (18 × 13.5 × 4.5 cm). The water was changed every day, and the larvae were fed a diet of dog biscuits and hydrolyzed yeast (*w*/*w*). The breeding medium was inspected every day, and any dead larvae were disposed of. In order to prevent outside mosquitoes from contaminating the breeding pots, muslin cloth was used to cover them. Each day, 500 mL of water was added to glass beakers containing larvae and pupae, as reported by Kamaraj et al. [22]. Pupae were put in a mesh cage (30 × 30 × 30 cm) and moved from the emergence into a separate jar of distilled water. Cotton pads soaked in a 10% glucose solution were used to keep the adult mosquitoes in a cage. The adult cage egg-laying medium was a cup of distilled water. When it came time to hatch the eggs, they were put in a clean, distilled water tray. The technique continued until a sufficient number of adult mosquitoes were obtained for the repellence bioassays.

### 2.4. Herbal Essential Oil Formulation (EOF)

The extracted EOs were combined to make 10 mL and then ultrasonically processed for 10 min. The extracted oils included 0.25 mL of *C. nardus* leaf oil, 0.25 mL of *E. globulus* leaf oil, 0.25 mL of *C. limon* fruit peel oil, 4.25 mL of *T. ammi* seed oil, and 5 mL of *M. arvensis* leaf oil. The toxicity of the mosquito larvae served as the basis for standardizing the herbal formulation. All of the bioassay activities used the same formulation.

### 2.5. Larvicidal and Pupicidal Bioassay

The larvicidal potential of a herbal essential oil formulation (EOF) and its active constituents was assessed against AA, AS, and CQ. DMSO (stock solution) was used to dissolve EOF in 100 mL of milli-Q water, which was then ultrasonically sonicated for 15 min. Then, 100–6.25 ppm of the standard was made using dechlorinated tap water. The final test solution contained DMSO (Qualigens) as an emulsifier at a concentration of 0.05%. The larvicidal and pupicidal activities were evaluated using the method employed by Kamaraj et al. [14,22,23] and minor modifications to the WHO procedure [24]. The larvae and pupa were collected for the toxicity test in five separate groups of 20, each containing 250 mL of water and the following EOF: 100, 50, 25, 12.5 and 6.25 ppm concentrations. DMSO and tap water were used as a control. The dead larvae and pupa were calculated after a 24 h period, and the mortality rate was calculated using the average of five repeats. The bioassay solution in which 100% of the larvae and pupa died on their own was chosen for the dose–response bioassay. To adjust the crude mortality%, Abbot’s formula was employed: {Percentage mortality = Number of died larvae/Number of live larvae introduced × 100}. 

### 2.6. Dose-Responsive Bioassay

Different concentrations of the standard solution’s larvicidal, pupicidal, ovicidal, oviposition-deterrent, and repellent characteristics were evaluated with 100 to 6.25 ppm concentrations. According to the preliminary testing results, EOF was put through a dose–response bioassay action against the larvae and pupae of AA, AS, and CQ. The number of dead larvae and pupa was recorded after 24 h of observations, and the mortality rate was computed using the average of five repeats. However, EOF samples proved to have identical harmful potential after 24 h.

### 2.7. Ovicidal Bio-Assay

By placing ovitraps in mosquito cages, the freshly laid eggs were retrieved for ovicidal activity. The ovitraps filter paper lining was used to lay the eggs. In total, 100 gravid females were allowed into the rearing cages with six oviposition cups 30 min before the start of the sunset period after scoring. The test solution’s EOF extract was added to five of the six cups at concentrations of 6.25, 12.5, 25, 50, and 100 ppm each, while DMSO and water served as a control. A minimum of 100 eggs were used for each treatment during the five times wherein the experiment was performed. After being cleaned and counted under a microscope, the eggs were treated, sieved through muslin cloth, and properly cleaned with tap water before being placed in plastic cubes filled with tap water for hatching evaluation in accordance with the methods developed by Su and Mulla [25] and Elango et al. [26]. The % egg hatching was estimated on the non-hatchability of eggs with closed opercula [27]. According to Rajkumar and Jebanesan’s method [28], the hatching rate of the eggs was assessed 98 h after treatment.

### 2.8. Oviposition-Deterrent Bio-Assay

The number of eggs laid in its presence and the efficiency of EOF as an oviposition deterrent were examined. Depending on the size of the pupae, a total of 40 adult insects, divided into 20 males and 20 females, were kept in 45 × 45 × 40 cm rearing cages with a 14:10 h light/dark cycle, 25 °C, and a relative humidity of 75–85%. The pupae were allowed to mature into adults inside the experimental environments. A 10% glucose solution was routinely administered to the adult population in disposable cups with cotton filaments. Each cage had five cups containing an oviposition filter, 9 cm of white filter paper, and 100 mL of distilled water [29]. In addition, 6, 12.5, 25, 50, and 100 ppm of EOF were included in each cup. So that we could have control, another cup was brewed without the EOF. Water and DMSO were used in the control setup. In order to eliminate any influence that location might have had on oviposition, the placements of the disposable cups were switched about among the five replicates. For each bioassay, cages were positioned next to one another in five repeats for each concentration. Following 24 h, a microscope was used to count the number of eggs that had been deposited in the treatment and control cups. The following formula determined the % actual repellency for each tested concentration.

$$ER\%\frac{NC-NT}{NC}\times 100$$

where ER stands for “effective repellency,” NC for “no control eggs,” and NT for “no treatment eggs” [30]. The results of the oviposition tests were expressed as the average number of eggs and the oviposition activity index (OAI), which was calculated using the equation below.

$$OAI=\frac{NT-NS}{NT+NS}$$

While there are NS, total eggs in the standard solution, there are NT, total eggs in the test solution. Oviposition active index values of at least +0.3 are considered attractants, whereas values of at least −0.3 are considered repellents [30]. Promising effects highlight the attraction of the test solutions and that more eggs were laid in the test cups than in the control cups. On the other hand, negative statistical evidence shows that the test solutions were a deterrent and that more eggs were dropped into the control cups than the test cups.

### 2.9. Repellency Test

Repellent bioassays were carried out according to Kamaraj et al. [23] and the WHO guidelines [29]. Laboratory-reared and pathogen-free mosquitoes of the AA, AS, and CQ species were transferred from the insect-rearing cage to test insect-repellent activity. To make test solutions of 100 ppm, the EOF was diluted with DMSO and Milli-Q water. Fifty blood-starved adult female mosquitoes aged 3 to 10 days from the laboratory were placed into individual cages measuring (45 × 45 × 40 cm) for the repellent experiment. A volunteer’s forearm and hand were cleaned with neutral unscented soap before each test, completely rinsed, and allowed to dry for 10 min before applying EOF. The various EOF extract concentrations were applied anywhere between the elbow and the tips of the fingers. The arm was not touched. The control arm received DMSO treatment, and the positive control arm received 1% DEET solution in ethanol. The cage received both the untreated and treated arms at the same time. For a period of 5 min, every 30 min from 3 h to 5 h, the number of landings was counted (efficacy in terms of landing) [29]. The protection time is the timeframe between when an EFO was applied and the final observational period before a bite was confirmed. The tests were stopped, and protection duration was recorded as 300 min if there were no confirmed bites after 300 min. A mosquito’s effort to stab its stylets into the skin was regarded as a bite. The test was repeated with a fresh batch of insects after the control arm’s experiment was terminated to demonstrate that the absence of bites was caused by repellence and not because the mosquitoes were not attracted to blood meals at the time. It was noticed that the EOF extract did not irritate the surface of the skin [31,32].

Protection = ({Number of bites received by control arm} − {Number of bites received by treated arm})/(Number of bites received by control arm) × 100.

### 2.10. Chemical Analysis of EOF by GC-MS

GC-MS analysis was used to identify the chemical components in the EOF extract according to the investigation of Kamaraj et al. [14]. This experiment inserted a 1 µL sample onto a 5 MS column of the GC-MS model using helium as the mobile phase (PerkinElmer, Clarus 500, Waltham, MA, USA). The samples were assessed subjectively and quantitatively using CP 3800 Saturn 2200 GC-MS equipment. The program’s temperature started at 80 °C and gradually increased to 350 °C by adding 3 °C every minute. A scan range of 20–500 AMU was used, and the ion temperature was set at 200 °C (Atomic Mass Unit). The relevant chemicals’ mass spectra library was compared to the peak attributes of the solvent fractions with respect to the mass-to-charge ratio.

### 2.11. Molecular Docking

Protein–ligand docking associated with the input to CB-Dock was used for molecular docking [33]. PDB: 3Q8I was selected for this investigation since it contains the crystal structure of the *Anopheles gambiae* odorant-binding protein 4 in association with indole along with the following information: Resolution and non-mutant characteristics 2.00 Å. N,N-Diethyl-meta-toluamide (DEET) was employed as a control to compare the docking score. DEET is the most effective, widely used, and well-established insect repellent [34].

### 2.12. Statistical Evaluation

SPSS software (Version 23) was used to conduct all of the data analyses, with a significance level set at a Type I error (*p*) value of less than 0.05. The assumptions necessary to employ parametric models were first applied to all of the obtained data. The mortality index of AA, AS, and CQ larvae and pupa exposed to the EOF extract was examined using the covariates “concentration” and “larval and pupal stage” (IV instar larvae and pupa) as well as the two-way ANOVA (with Tukey post-test) (five levels: 6.25, 12.5, 25, 50, and 100 ppm). Regression and correlation analyses were also performed. We used a two-way ANOVA (with Tukey post-test) to examine the impact of the chemicals added to the exposure media on the death rate of all three mosquito species while accounting for the “exposure” components (for each larval and pupal stage). Larvae and pupae on two levels and concentrations (6.25, 12.5, 25, 50, and 100 ppm) were used. By utilizing probit analysis to plot the mortality index versus the concentrations of the EOF extract, it was used to calculate the 24 h lethal concentrations (LC_50_).

## 3. Results

### 3.1. Essential Oil Production 

The EO content of the *T. ammi* seeds was the highest of the five plant species employed in the current investigation, whereas the material from *M. arvensis* yielded the least quantity of steam-distilled EOs (Table 1). Similar amounts of EOs were found in the plant material from *C. nardus* and *E. globulus* species, though they were less abundant than those from *T. ammi* and *C. limon*, and their percentage yields were higher than those obtained from *M. arvensis*. These essential oil concentrations were lower than those in *T. ammi* and *C. limon* (Table 1).

### 3.2. Larvicidal and Pupicidal Bioassay

**Figure 2:**
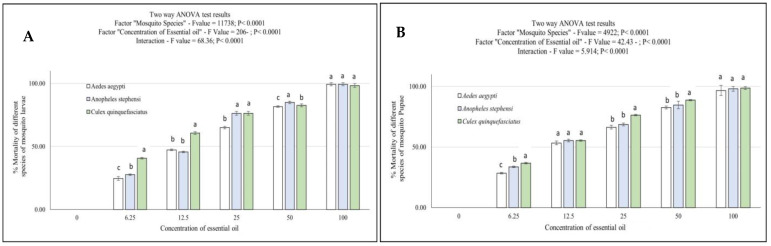
Mortality index of *Ae. aegypti*, *An. Stephensi*, and *Cx. quinquefasciatus* 4th instar larvae and pupa [(**A**) larvae and (**B**) Pupa] exposed to the essential oil formulation. Bars indicate the mean ± SD. The data were subjected to a two-way ANOVA with a 5% chance of success for the Tukey post-test. At the top of the charts are statistical summaries. Differences between groups exposed to each type of exposure concentration are indicated by distinct lowercase letters. The concentrations to which *Ae. aegypti*, *An. stephensi*, and *Cx. quinquefasciatus* larvae and pupa were exposed (each test was repeated at *n* = 5), corresponding to the concentrations at which small letters show significant differences between groups treated with essential oil at 6.25, 12.5, 25, 50, and 100 ppm.

**Figure 3:**
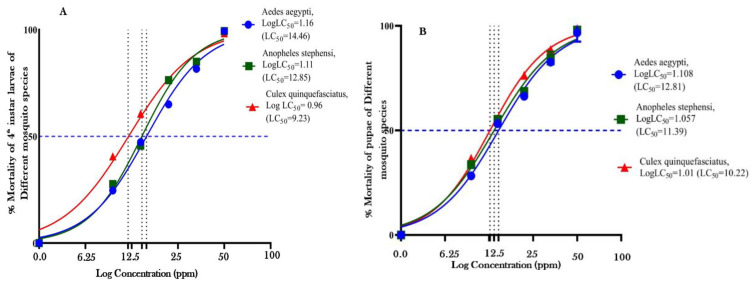
*Ae. aegypti*, *An. stephensi*, and *Cx. quinquefasciatus* 4th instar larvae and pupa [(**A**) Larvae and (**B**) Pupa] in response to varying concentrations of EOF, and a mortality curve was generated using the concentration–response (Probit) model.

The EOFs’ 24-h larvicidal and pupicidal efficacies on three mosquito species were assessed at doses of 6.25, 12.5, 25, 50, and 100 ppm. Figure 2A,B displays the % mortality as well as the minimal fatal concentration. The evaluation of larvicidal efficacy showed that the EOF had good larvicidal effects on mosquito vectors. While promising pupicidal action was seen in 24 h concentrations of 50 and 100 ppm, the maximum mortality of 100% was reported for all three mosquito larvae and pupa at 24 h of 100 ppm for AA, AS, and CQ (Figure 2A,B). The larvicidal activities of the EOF were *Ae. aegypti* (LC_50_ = 14.46, 95% CI = 13.34 and R^2^ value 0.990), *An. stephensi* (LC_50_ = 12.85, 95% CI = 13.80 and R^2^ value 0.992) and *Cx. quinquefasciatus* (LC_50_ = 9.232, 95% CI = 10.31 and R^2^ value 0.984 ppm), respectively Figure 3A at 24 h exposure. Similarly, pupicidal activities were (LC_50_ = 12.81, 95% CI = 14.03 and R^2^ value 0.988) shown in *Ae. aegypti*, (LC_50_ = 11.39, 95% CI = 12.49 and R^2^ value 0.988) observed in *An. stephensi* as well as (LC_50_ = 10.22, 95% CI = 10.89 and R^2^ value 0.994) and *Cx. quinquefasciatus* Figure 3B and Table 2. Hence, no mortality was observed in the DMSO controls. The concentration–response (Probit) LC_50_ larval mortality curve for AA, AS, and CQ IV instar larvae and pupa are presented in Figure 3A,B.

In the present investigation, we found no significant interaction between the parameters “larval and pupal stage” and “concentration” in the larvicidal and pupicidal potential of the EOFs (Figure 2A,B). Likewise, concerning the results attained for the EOF extracts in terms of larvicidal and pupicidal effects, the factor “concentration” was accountable for >98% of the total discrepancy in larvae (F-value = 206; *p* < 0.0001 and interaction F-value = 68.36; *p* < 0.0001) and pupa (F-value = 42.43; *p* < 0.0001 and interaction F-value = 5.914; *p* < 0.0001), subsequently, the experiment was considered greatly significant in the assessment of the toxicity of herbal EOFs. The data were analyzed using two-way ANOVA despite the strong relationships between the variables examined (larval and pupal stages) and a linear intensification in the harmfulness of EOFs when their concentrations were increased. The data are represented in Figure 2A.

### 3.3. Ovicidal Effect of EOF 

**Figure 4:**
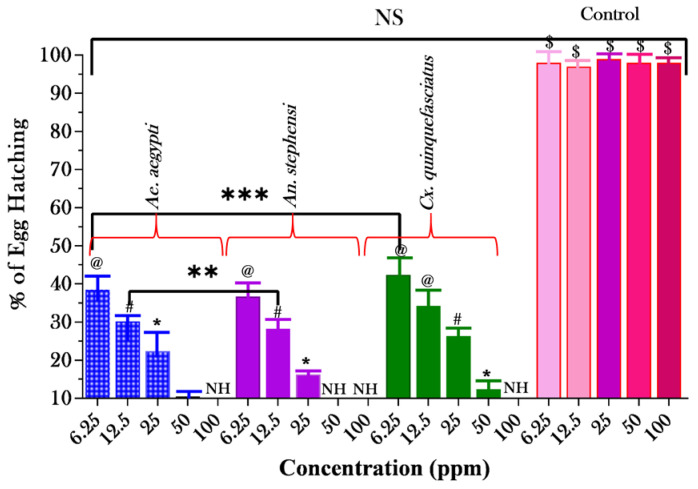
Different concentrations of an essential oil formulation (EOF) have been shown to have mosquito ovicidal activity against the eggs of *Ae. aegypti*, *An. stephensi*, and *Cx. quinquefasciatus*. (*, @, # and $) Different letters on the bars showed significant differences when the egg hatching was compared between different test concentrations after a particular time period separately (*p* < 0.05). Using only tap water as the control, 100% of the eggs hatched. Repeating the experiment with n = 5 and NH (no hatchability), resulted in 100% mortality (**, *** Significant in each tested groups; NS: non-significant in tested and control groups).

The egg hatchability (%) of AA, AS, and CQ with the EOF used are presented in Figure 4. The highest 100% no egg hatchability at the highest concentration of 100 ppm and low egg hatchability of 38 ± 2.34, 36 ± 1.74, and 42 ± 1.39% were observed in a lower concentration of 6.25 ppm against AA, AS, and CQ. The concentration of the herbal EOF was directly connected with the % of egg hatchability, which was proportional to the number of eggs. According to the current study’s findings, 100% egg no hatchability was noted in the EOF extract at 100 ppm. In the control studies (untreated), a hatchability of almost 100% was attained. 

### 3.4. Oviposition-Deterrent Potential of EOF 

In the oviposition deterrent experiment, gravid females of AA, AS, and CQ chose to lay their eggs in the distilled water control cups as opposed to the cups that had been treated with EOF and DMSO (Table 3). Additionally, the current findings revealed that the concentration of 100 ppm treated cups averaged 16 ± 1.56, 15 ± 1.19, and 31 ± 1.26% eggs laid per cup containing the EOF extract, whereas the control cups averaged 410 ± 1.61, 565 ± 1.60, and 360 ± 1.64% eggs laid per cup. When compared to the control solutions, a considerably lower percentage of eggs are laid on ovitraps encompassing the EOF extracts (*p* < 0.05). The EOF extracts effectively dispirited oviposition by the gravid mosquitoes. The highest rates of effective repellency against oviposition were 96.09, 94.83, and 91.39%, respectively, recorded at 100 ppm. At 100 ppm, the EOF extracts obtained OAI values of −0.92, −0.95, and −0.84 correspondingly. The OAI values demonstrated the deterring impact of the EOF extracts, which resulted in a strikingly negative reaction and very few eggs being laid (Table 3).

### 3.5. Repellent Effect: Laboratory Evaluation

**Figure 5:**
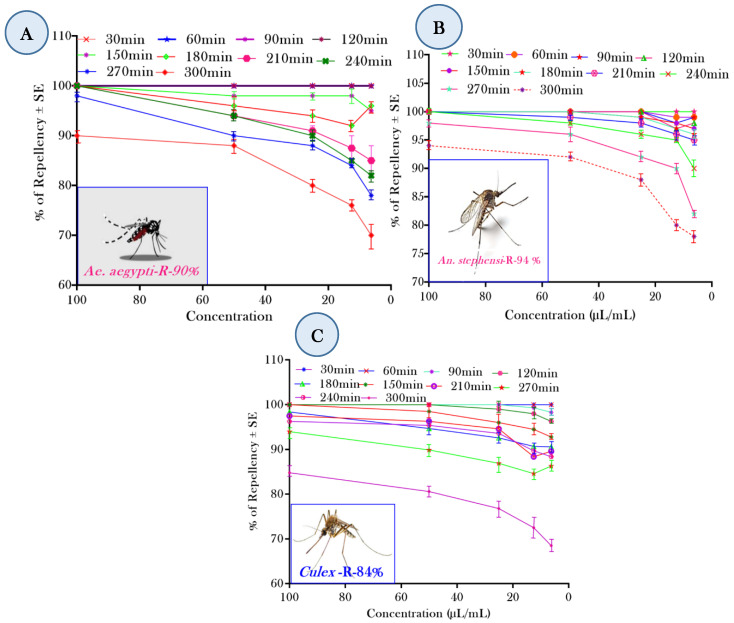
The effectiveness of varying concentrations of EOF and DEET in terms of repellence against (**A**) *Ae. aegypti*, (**B**) *An. stephensi*, and (**C**) *Cx. quinquefasciatus* mosquitoes. Time protection and test concentration varied considerably (*p* < 0.05). The standard deviation is shown by the error bars on each line.

The repellent effect of the herbal EO-based formulations in the laboratory condition is shown in Figure 5A–C. For all the concentrations tested, the formulation of HOF demonstrated 5 h of 100% repellent effect against the AA, AS, and CQ mosquitoes. At the highest concentration (100 ppm), >90% of the repellent effect was maintained up to 5 h post-application, while at a 6.25 ppm concentration, a >90% repellent effect was maintained up to 3 h against *Ae. aegypti*, 96% and >80% repellent effect for 5 h post-application in terms of *An. stephensi* and *Cx. quinquefasciatus* was observed at 100 ppm. For herbal oil formulations at 100 ppm concentrations, a 100% repellent protection effect was observed in the 3 h post-application against three mosquito species; following 4 h, the repellent effect only reduced slightly to 90 ± 1.68%, 96 ± 1.20%, and 80 ± 1.46%, respectively, at 100 ppm concentrations, respectively. More than an 80% repellent effect was observed up to 6 h post-application, which after 8 h, decreased to no less than 75% at 100 ppm concentration, and a 100% repellent effect was observed for 4 h against *Ae. aegypti* and *An. stephensi*. 

### 3.6. GC-MS Analysis of EOF

**Figure 6:**
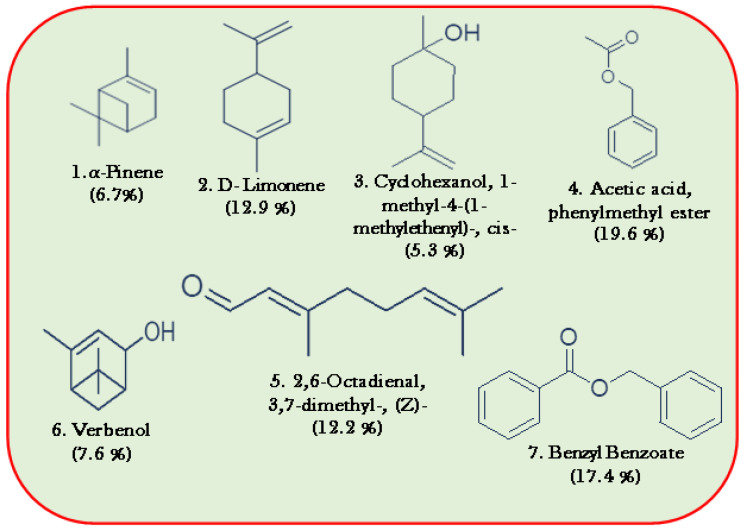
Major chemical constituents of EOFs.

A total of 34 distinct compounds were found in the five formulations of the most bioactive EOs, as evaluated via GC-MS (Table 4). EOFs rich in α-pinene (6.7%), D-limonene (12.9%), cyclohexane, 1-methyl-4-(1-methylethenyl)-, *cis*-(5.3%), acetic acid, phenylmethyl ester (19.6%), 2,6-octadienal, 3,7-dimethyl-, (Z)-(12.2%), verbenol (7.6%), benzyl benzoate (17.4%) are presented in Figure 6. The minimum level, such as γ-terpinene (1.6%), 2-pentanone, 4-(2,6,6-trimethyl-2-cyclohexenyl) (2.3%), geranyl acetate (1.1%), 1h-benzocycloheptene, 2,4a,5,6,7,8,9,9a-octahydro-3,5,5-trimethyl-9-methylene (1.0%), isoaromadendrene epoxide (1.0%), and di-epi-α-cedrene-(I) (2.7%) were present in the EOFs. Finally, d-limonene, as well as 2,6-octadienal, 3,7-dimethyl-, (Z)-, including benzyl benzoate, were the main constituent of the EOFs, and all of the compounds constituted 99.998% of the combined list of EOF compounds tabulated in Table 4.

### 3.7. Molecular Docking Analysis of OBP4

**Figure 7:**
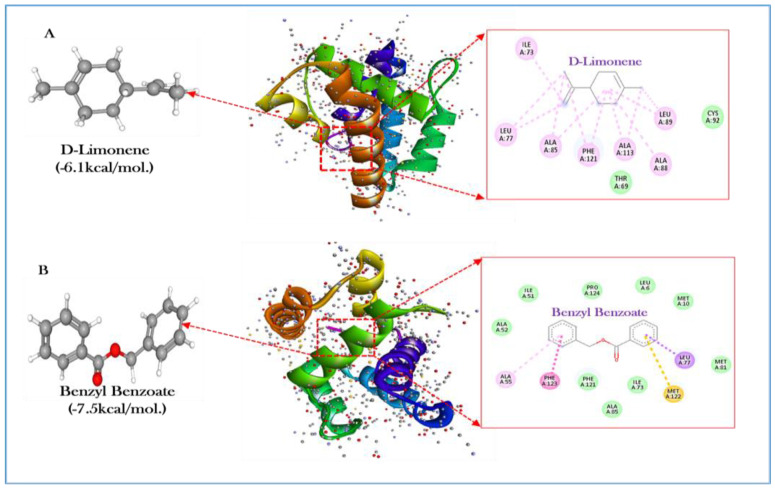
Two-dimensional and three-dimensional intermolecular contact between EOF compounds (**A**) D-limonene and (**B**) Benzyl benzoate against mosquito odorant-binding protein 4 (PDB: 3Q8I).

In the current study, we used a structure- and ligand-based virtual screening strategy, beginning with seven main chemicals from five plants derived through the extraction of EOs. The molecular docking report suggests that d-limonene (−6.1 kcal/mol) and benzyl benzoate (−7.5 kcal/mol) have significantly similar binding affinities as the positive control n,n-diethyl-meta-toluamide (−6.3 kcal/mol). The amino acid residues are the gate channel openers in the active sites, which affirms the therapeutic activity of d-limonene and benzyl benzoate. Two-dimensional and three-dimensional intermolecular contact between EOF compounds against mosquito odorant-binding protein 4 (PDB: 3Q8I) is presented in Figure 7A,B. The molecular binding scores of EOFs’ major seven compounds with amino acid residues are tabulated in Table 5. By simulating the interaction between a minor molecule and a protein at the atomic level, the molecular docking method enables us to characterize how small molecules interact with target proteins’ binding sites and better comprehend the fundamental biological processes.

## 4. Discussion

In most tropical and subtropical nations, vector-borne diseases are the main source of morbidity, and they have traditionally presented as a challenge to medical professionals working to improve human welfare. For a number of these disease-causing pathogens, mosquitoes are the most lethal vector. The current study assessed the effectiveness of herbal EOF in controlling larvae and pupae and acting as a potential repellent of AA, AS, and CQ adults in a period during which insecticide resistance to synthetic chemical pesticides is increasing.

The mosquito species vectors are incredibly successful due to their capacity to adapt to a wide range of climatic circumstances [35,36,37,38]. Since the 1980s, public health initiatives have used the organophosphate substance temephos in potable water as the most popular and effective anti-larval treatment. Nevertheless, at the moment, emergeing and increasing temephos resistance in nations such as Brazil and Thailand have been reported [39,40]. Ae. aegypti resistance to temephos has not previously been reported in India, but recent discoveries from the Andaman and Nicobar Islands, Assam, and Tamil Nadu raise the alarm in India as well [41,42]. Natural remedies made from plants have long been used as insect repellents in various parts of the world, mainly to stop obnoxious biting [43]. The current research was concentrated on natural plant products since they could offer the benefit of being environmentally beneficial. Additionally, our results support the utilization of EOs due to their larvicidal and repulsive properties. Thymol, carvacrol, and α-pinene showed the most toxic activity against IV-instar larvae of Cx. Pipiens, with LC_50_ = 36–49 mg/L [44]. Similarly, the hydrodistilled E. globulus EO exhibited promising larvicidal potential against AS, with LC_50_ of 30.19 ppm and LC_90_ values of 103.38 ppm; Ae. aegypt: 13.57 ppm and 106.75 ppm; and Cx. quinquefasciatus: 7.46 ppm and 32.45 ppm at 24 and 48 treatments [45].

Interestingly, the proposed herbal EOF showed a greater larvicidal effect on three mosquito larvae of AA (LC_50_ = 14.46 μL/mL), AS (LC_50_ = 12.85, μL/mL), and CQ (LC_50_ = 9.232, μL/mL), respectively; α-pinene (6.728%) was a major constituent. Pugazhvendan and Elumali [30] have tested three different plant EOs from *Cinnamomum camphora*, *Myrtus caryophyllus*, and *Eucalyptus globulus*, which showed excellent larvicidal potential in terms of AA, AS, and CQ, with LC_50_ values of 68.18 and LC_90_ values of 248.37 ppm against *Aedes*, respectively. Accordingly, a previous researcher, Manimaran et al. [46], reported the larvicidal activities of calamus, cinnamon, citronella, clove, eucalyptus, lemon, mentha, and orange oils, which showed 100% at 1000 ppm, while the knockdown effect was observed to be 100% at 10 ppm against AA, AS, and CQ, respectively. Correspondingly, the lethal toxicity LC_50_ values of 39.74 and 115.67 ppm and LC_90_ 46.23 and 165.36 ppm, respectively, were observed for mentha oil against AS and AA. Similar results were obtained by Medhi et al. [47] when they used 160 ppm of Eucalyptus oil on AS larvae. It iss interesting to observe the diversity in terms of the LC_50_ values between investigations, which may be explained by the various oil extraction methods or formulas employed by the various manufacturers. Since they create a monomolecular film on the water’s surface, herbal EOFs have larvicidal, pupicidal, and ovicidal effects. The film lowers the aqueous layer’s surface tension, which kills larvae and pupae by obstructing the spiracular opening and preventing tracheal respiration. Accordingly, we found that the efficacy of the EOF was consistent across different volumes of water bodies with the same surface area because it could generate a homogeneous layer on the water’s surface regardless of the volume. Oils’ physicochemical properties, such as specific gravity, surface tension, and viscosity, can cause them to spread horizontally across a smooth and slippery surface. Therefore, the development of a uniform oily layer on the surface likely boosted the effectiveness of larvicidal and pupicidal activities.

The findings demonstrated that, even at lower concentrations, the essential oil formulation from T. ammi, M. arvensis, C. nardus, E. globulus, and C. limon could repel or prevent Ae. aegypti, An. stephensi, and Cx. quinquefasciatus mosquitoes from biting. The term “insect repellent” is used for a synthetic chemical or natural agent that induces insects to flee from its source [48]. DEET, which prevents mosquitoes and other biting insects, is quite effective [49,50]. Conversely, research by Robbins and Cherniack [51] and Qiu et al. [52] evidenced that the severity of the human toxicological response to DEET application varies from mild to severe. Researchers have undertaken significant research on herbal repellents in order to avoid the hazardous effects of DEET. As a result, attention is now directed at developing natural repellents with environmental care [53,54,55,56].

Since many plants are excellent sources of aromas that repel mosquitoes and other arthropods, including herbivores, herbal EOs are desirable insect repellents [57]. Traditional sources of natural insect repellents, including Eucalyptus and Citronella EOs, are well-known [58]. Acyclic or monocyclic monoterpenes, which are prevalent in EOs, have been observed to have spatial-repellent capabilities; for instance, insect sensilla are capable of detecting volatile monoterpenes [59]. The applied repellents create a vapour barrier that keeps insects from landing on the surface and biting [60]. Our investigation demonstrated the excellent repellent capabilities of producing five plant-based EOF against AA, AS, and CQ mosquitos, resulting in zero mosquito landing for 5 h. Remarkably, the proposed EOF showed greater larvicidal effects on three mosquito larvae, in particular against AA, with LC_50_ values of 14.46 ppm, against AS, with LC_50_ values of 12.85, ppm and against CQ, with LC_50_ values of 9.232 ppm, respectively, with α-pinene (6.728%) as the major constituent. The three main categories of monoterpenoids, sesquiterpenoids, and phenylpropanoids make up most of the chemical components of T. ammi (Apiaceae) EOs, exhibiting the strongest insecticidal activity. Monoterpenoids can cause neurotoxicity against mosquitoes [61]. Sarma et al. [62] studied the mosquito larvicidal, ovicidal, and adulticidal potential of *α* and *β*-pinenes against Ae. aegypti. β-pinene showed higher toxicity than α-pinene against egg and larvae with LC_50_ values of 21.2 and 108.4 ppm at 72-h, respectively. Milugo et al. [63] observed that the root Parthenium hysterophorus extract and its constituents β-phellandrene, α-phellandrene, α-pinene, (E)-caryophyllene, and 3-carene inhibited the ovicidal effect against water-attracted gravid females of An. gambiae and the minimum level of eggs hatched (55%) at lower concentrations of 0.25 to 4 µg/µL compared to the control water (66%). Furthermore, d-limonene inhibits eggs and larvae and exerts fumigant toxicity on stored product beetles and grain pests in empty godowns, with potential ovicidal and feeding-deterrent actions [64]. As Sharma et al. [65] have reported previously, EOs extracted from C. reticulate, C. limon, and C. limetta (peels) have shown larvicidal activity with LC_50_ values of 16.31 and 3.50 and LC_90_ values of 77.60 and 18.17 ppm, and LC_50_ values of 29.20 and 3.11 ppm and LC_90_ values of 68.62 and 9.03 ppm, respectively, against AS and CQ after 24 h. According to the GC-MS data, the Citrus leaves and peels contain alkaloids, terpenoids, anthraquinones, flavonoids, and tannins. Citral, pinene, terpinene, linalool, and d-limonene were identified as being major phytocompounds.

According to Soonwera et al. [66], limonene and trans-anethole were the two main components of EOs derived from the Illicium verum and Zanthoxylum limonella, respectively, which showed potential activity larvae and pupae of Ae. aegypti and Ae. Albopictus, with the LT_50_ values ranging from 0.2 to 6.9 h, and the I. verum EOs + trans-anethole (2.5%) combination having excellent activity against larvae and pupae. Compared to the two mosquito species examined, trans-anethole showed greater larvicidal and pupicidal activity (LC_50_ ranging from 2.4 to 3.4%) than d-limonene (LC_50_ ranging from 2.5 to 3.7%). There have been reports of strong efficacy concerning d-limonene and α -pinene against a wide range of insect pests. D-limonene and α-pinene have been shown to be highly effective, as evidenced by the following papers: stronger larvicidal activity against Ae. albopictus was reported by Seo et al. [67]; good oviposition-deterrent effectiveness against Ae. aegypti was reported by Waliwitiya et al. [68]; and promising larvicidal activity against Ae. aegypti was reported by Pandiyan et al. with LC_50_ of 50.2 mg/L^−1^ [69]. A study conducted by Aungtikun et al. [70] confirmed that trans-anethole showed a strong insecticidal effect on adult Musca domestica. The findings of this investigation validated the papers mentioned above and showed that these two substances were extremely harmful to the larvae and pupae of AA, AS, and CQ.

According to their experimental findings, EOFs in optimal concentrations are safe for the environment, harmless to fish and plankton, swiftly disintegrate in the environment, and are not hazardous to people or other mammals. The possibility that insects could develop resistance to EOs is raised because no investigation on the five plant formulation products has yet shown any indication of emerging insect resistance to any EOF. This advantageous characteristic is probably due to the fact that EOFs have a variety of modes by which they damage insects [71]. By the way, the existence of d-limonene and α-pinene in this study may be explained by multiple identical mechanisms of action. According to Dhinakaran et al. [72], d-limonene interferes with the physiological responses of insects to Ae. aegypti by down-regulating the enzymes acetylcholinesterase (AChE) and butyrylcholinesterase (BChE), which disrupts the insect’s endocrine system while it is going through its moulting process.

In the present study, the EOF compounds of α-pinene, d-limonene, β-citral, acetic acid phenylmethyl ester, 2,6-Octadienal, 3,7-dimethyl-(Z)-,verbenol, and benzyl benzoate showed satisfactory structural similarity with DEET (−6.3 kcal/mol) according to the following binding affinity values, −5.9, −6.1, −5.8, −5.6, −5.7, −5.2, and −7.5 kcal/mol, respectively. Supporting this high structural similarity, d-limonene and benzyl benzoate also displayed an advantageous physicochemical association with conventional topical repellents (Table 5). Interestingly, numerous studies have emphasized the insect-repellent qualities of EOs and thymol acetate, which showed repellent potential in terms of crop pest Meligethes aeneus (Fabricius) (Coleoptera: Nitidulidae) and the mosquito disease vectors of An. subpictus and An. stephensi [73]. The monoterpene derivative of carvacrol, known as carvacryl acetate, which is observed to have excellent repellent properties in high concentrations in the EOs of some Lamiaceae species, including Clinopodium sp. [74] and Thymus sp. [75], has been shown to have insecticidal and oviposition deterrent activities against a diversity of insect species [76,77]. Furthermore, p-cymen-8-ol and its derivative p-cymene have been utilized to treat An. gambiae [78] and Lasioderma serricorne Fabricius (Coleoptera: Anobiidae), demonstrating excellent repellent activity [79,80].

Additionally, previous investigations have reported that natural products, such as alcohols and sesquiterpenes, have shown particular inhibitory effects against insect OBPs [81]. Concerning the effectiveness of varying concentrations of EOF, we found that compounds such as d-limonene and benzyl benzoate simulate the binding mechanism of topical repellent DEET by sharing comparable pharmacophoric groups and intermolecular interactions with the protein pocket. The results of the effectiveness of varying concentrations of EOF supports earlier studies on the experimental repellency of this chemical class and sheds light on the molecular mechanism underlying these compounds’ resistance to insect olfactory identification [80,82,83,84]. Our results confirm the the potential of EOFs as an interesting and cost-effective source for manufacturing cutting-edge insect repellents.

## 5. Conclusions

The current study conclusively demonstrated that herbal EOFs were more active than commercial products used as topical repellents for controlling Ae. aegypti, An. stephensi, and Cx. quinquefasciatus mosquito vectors. They were also effective larvicidal, pupicidal, ovicidal, oviposition deterrent, and repellent agents. The current mosquito population’s heightened resistance to synthetic pesticides could contribute to obtaining higher efficacy. More importantly, as the five plants have been used in India for food and traditional medicine since ancient times, it may be considered that the herbal EOF alternatives are far safer for people and the environment than synthetic ones. It is possible that a commercial insecticidal product based on the herbal EOF called “Mosquito Repellent Oil” would one day replace synthetic pesticides.

## Figures and Tables

**Table 1:** The proportion of essential oils that were successfully extracted from freshly cut aerial parts, fruit peel, and plant seeds.

Name of the Plants	Family Name	Essential Oil Yield (%)
*Cymbopogon nardus*—Leaves	Poaceae	0.54
*Eucalyptus globulus*—Leaves	Myrtaceae	0.47
*Mentha arvensis*—Leaves	Lamiaceae	0.36
*Citrus limon*—Fruit peel	Rutaceae	0.58
*Trachyspermum ammi*—Seeds	Apiaceae	0.74

**Table 2:** The essential oil extract’s LC_50_ against the *Ae. aegypti*, *An. stephensi*, and *Cx. quinquefasciatus larvae and papae*.

Stages	Species of Mosquito	Best Fit Values		95% CI		Goodness of Fit	
		Log LC_50_	LC_50_	LogLC_50_	LC_50_	DF	R Square
Larvae	*Ae. aegypti*	1.16	14.46	1.125–1.195	13.34–15.66	16	0.990
	*An. stephensi*	1.109	12.85	1.077–1.140	11.95–13.80	16	0.992
	*Cx. quinquefasciatus*	0.965	9.23	0.915–1.013	8.228–10.31	16	0.984
Pupae	*Ae. aegypti*	1.108	12.81	1.067–1.147	11.68–14.03	16	0.988
	*An. stephensi*	1.057	11.39	1.015–1.097	10.35–12.49	16	0.988
	*Cx. quinquefasciatus*	1.01	10.22	0.9813–1.037	9.579–10.89	16	0.994

Control—No mortality. LC_50_, lethal concentration; CI, confidence interval; DF, degree of freedom.

**Table 3:** Oviposition deterrence of EOF extract against *Ae. Aegypti*, *An. Stephensi*, and *Cx. quinquefasciatus*.

Mosquito Species	Concentration (µL/mL)	Number of Eggs ± SE		Effective Repellency (%)	Oviposition Active Index
		Treated	Control		
*Ae. aegypti*	100	16 ± 1.56 ^aa^	410 ± 1.61	96.09 ^aa^	−0.92
	50	54 ± 1.12 ^bb^	480 ± 1.34	88.75 ^bb^	−0.79
	25	96 ± 1.44	340 ± 1.16	71.76 ^cc^	−0.56
	12.5	76 ± 1.26 ^ee^	196 ± 2.16	62.22 ^dd^	−0.44
	6.25	74 ± 2.28 ^ee^	180 ± 1.39	58.89 ^ee^	−0.42
*An. stephensi*	100	15 ± 1.19 ^aa^	565 ± 1.60	94.83 ^aa^	−0.95
	50	44 ± 1.26 ^cc^	360 ± 1.61	87.77 ^bb^	−0.78
	25	56 ± 1.34 ^bb^	340 ± 1.20	83.53 ^bb^	−0.72
	12.5	62 ± 1.14 ^dd^	280 ± 1.17	77.86 ^cc^	−0.64
	6.25	68 ± 1.62	190 ± 1.21	64.42 ^dd^	−0.43
*Cx. quinquefasciatus*	100	31 ± 1.26	360 ± 1.64	91.39 ^aa^	−0.84
	50	42 ± 1.34 ^cc^	352 ± 1.56	88.07 ^bb^	−0.78
	25	62 ± 1.17 ^dd^	430 ± 1.30	85.58 ^bb^	−0.75
	12.5	72 ± 1.20 ^ee^	212 ± 1.16	66.98 ^dd^	−0.50
	6.25	80 ± 1.76	190 ± 1.53	57.89 ^ee^	−0.41

ANOVA and Duncan’s Multiple Range Test results show that the means of five replicates SE in each row versus each mosquito species followed by a different letter are significantly other (*p* < 0.05).

**Table 4:** Chemical composition of EOFs obtained via GC-MS analysis.

S. No	Retention Time	Compounds	Molecule Formula	Exact Mass Values	Peak Area %
1	3.989	α-Pinene	C_10_H_16_	136.1252	6.728
2	4.295	2-Carene	C_10_H_16_	136.1252	0.830
3	4.479	α-Terpineol	C_10_H_18_O	154.1357	0.583
4	4.620	D-Limonene	C_10_H_16_	136.1252	12.980
5	4.710	iso-β-terpineol	C_10_H_18_O	154.1357	5.356
6	4.771	Eucalyptol	C_10_H_18_O	154.1357	0.149
7	4.780	Trifluoro acetyl-α-terpineol	C_12_H_17_F_3_O_2_	250.1180	0.192
8	4.818	γ-Terpinene	C_10_H_16_	136.1252	1.663
9	4.945	3,4-Dimethylbenzyl alcohol	C_9_H_12_O	136.0888	0.276
10	5.006	(R)-linalool	C_10_H_18_O	154.1357	0.198
11	5.044	Limonene oxide, cis	C_10_H_16_O	152.1201	0.189
12	5.326	Acetic acid, phenylmethyl ester	C_9_H_10_O_2_	150.0680	19.688
13	5.529	Isoeugenol acetate	C_12_H_20_O_2_	196.1463	0.767
14	5.694	β-Citral	C_10_H_16_O	152.1201	12.288
15	5.807	Verbenol	C_10_H_16_O	152.1201	7.655
16	5.939	Thymol	C_10_H_14_O	150.1044	0.256
17	5.995	Dihydromethyl-alpha-ionone	C_14_H_24_O	208.1827	2.350
18	6.151	Geranyl acetate	C_12_H_20_O_2_	196.1463	1.139
19	6.362	Caryophyllene	C_15_H_24_	204.1878	0.529
20	6.424	Eremophylene	C_15_H_24_	204.1878	0.569
21	6.471	α-Himachalene	C_15_H_24_	204.1878	1.029
22	6.555	Isoaromadendrene epoxide	C_15_H_24_O	220.1827	1.008
23	6.631	Di-epi-α-cedrene-(I)	C_15_H_24_	204.1878	2.743
24	6.645	γ-Murolene	C_15_H_24_	204.1878	0.478
25	6.701	Bicyclo[4.1.0]heptan-2-ol	C_16_H_24_O_3_	264.1725	0.112
26	6.810	Valerenic acid	C_15_H_22_O_2_	234.1619	0.047
27	6.843	Calamene	C_15_H_22_	218.1670	0.038
28	6.890	Caryophyllene oxide	C_15_H_24_O	220.1827	0.374
29	6.970	Diepicedrene-1-oxide	C_15_H_24_O	220.1827	0.134
30	7.007	Calarene epoxide	C_15_H_24_O	220.1827	0.266
31	7.097	Cubenol	C_15_H_26_O	222.1983	0.544
32	7.153	Tumerone	C_15_H_22_O	218.1670	0.460
33	7.210	α-Cyperone	C_15_H_22_O	218.1670	0.892
34	7.497	Benzyl benzoate	C_14_H_12_O_2_	212.0837	17.488

**Table 5:** Molecular docking scores of seven major compounds against mosquito odorant-binding protein 4 (PDB: 3Q8I).

S. No.	Compounds	Binding Affinity PDB:3Q8I (kcal/mol)	Amino Acid Residues
1.	α-Pinene	−5.9	ALA48 ILE51 ALA52 ALA55 THR57 ILE64 THR69 ALA106 SER109 ALA110 PHE121 PHE123
2.	D-limonene	−6.1	HR69 ILE73 LEU77 ALA85 ALA88 LEU89 CYS92 SER109 ALA113 PHE121 MET122
3.	iso-β-terpineol	−5.8	ALA52 THR57 ILE64 THR69 LEU89 LYS105 ALA106 TYR107 SER109 ALA110 ALA113 PHE121 PHE123
4.	Acetic acid, phenylmethyl ester	−5.6	ILE64 THR69 ILE73 ALA85 ALA88 LEU89 ALA106 SER109 ALA110 ALA113 PHE121
5.	β-citral	−5.7	THR69 ILE73 LEU77 MET81 MET84 ALA85 ALA88 LEU89 ALA113 THR117 THR120 PHE121 MET122
6.	Verbenol	−5.2	ALA48 ALA52 THR57 ILE64 THR69 ALA106 TYR107 SER109 ALA110 PHE121 PHE123
7.	Benzyl Benzoate	−7.5	ALA48 ALA52 ILE64 THR69 ILE73 ALA85 ALA88 LEU89 CYS92 ALA106 SER109 ALA110 ALA113 PHE121 PHE123
8.	N,N-Diethyl-meta-toluamide (DEET) (Positive control)	−6.3	ALA52 THR57 ILE64 THR69 ILE73 ALA85 ALA88 LEU89 CYS92 ALA106 SER109 ALA110 ALA113 PHE121 PHE123

## Data Availability

The information used to support the study’ s findings can be obtained upon request from the corresponding author.

## Sample Availability

Samples of the compounds are available from the authors upon reasonable request.
